# Active Roles of Tumor Stroma in Breast Cancer Metastasis

**DOI:** 10.1155/2012/574025

**Published:** 2012-02-19

**Authors:** Zahraa I. Khamis, Ziad J. Sahab, Qing-Xiang Amy Sang

**Affiliations:** ^1^Department of Chemistry and Biochemistry and Institute of Molecular Biophysics, Florida State University, Tallahassee, FL 32306-4390, USA; ^2^Department of Oncology and Lombardi Comprehensive Cancer Center, Georgetown University Medical Center, Washington, DC 20007, USA

## Abstract

Metastasis is the major cause of death for breast cancer patients. Tumors are heterogenous cellular entities composed of cancer cells and cells of the microenvironment in which they reside. A reciprocal dynamic interaction occurs between the tumor cells and their surrounding stroma under physiological and pathological conditions. This tumor-host communication interface mediates the escape of tumor cells at the primary site, survival of circulating cancer cells in the vasculature, and growth of metastatic cancer at secondary site. Each step of the metastatic process is accompanied by recruitment of stromal cells from the microenvironment and production of unique array of growth factors and chemokines. Stromal microenvironment may play active roles in breast cancer metastasis. Elucidating the types of cells recruited and signal pathways involved in the crosstalk between tumor cells and stromal cells will help identify novel strategies for cotargeting cancer cells and tumor stromal cells to suppress metastasis and improve patient outcome.

## 1. Introduction

Breast cancer is the most common malignancy and the second major cause of mortality and morbidity in Western women [1]. The systemic outgrowth and spread of the cancer cells through a process known as metastasis is the main cause of deaths in these patients. Recently, disease-related mortality and metastasis have declined as a result of early diagnosis and application of adjuvant therapy. Mammographic screening, surgery, radiotherapy, chemotherapy, antibody therapy, and endocrine therapy facilitate the suppression of the metastatic dissemination of local tumor [2]. However, these treatments target the tumor cells and disregard the auxiliary cells present in the surrounding microenvironment that is also referred to as the stromal cells. These auxiliary cells, including myoepithelial cells, fibroblasts, myofibroblasts, endothelial cells, inflammatory cells, and bone-marrow-derived cells (BMDCs) such as macrophages, mast cells, neutrophils, and lymphocytes, are widely recognized to collaborate with cancerous cells and other host cells to create a tumor-permissive microenvironment capable of providing continuous support for tumor growth, progression, angiogenesis, invasion, and metastasis [3, 4].

Metastasis is the systemic dissemination of tumor cells at sites distinct from the primary lesion. It is a multistep process that involves detachment of cells from the primary tumor, followed by survival in the blood vessels or lymphatic system and finally development of secondary tumor. It is a poorly understood aspect of carcinogenesis that requires the clarification of the underlying cellular and molecular events that control the metastatic cascade from onset to colonization [5]. It is undisputed that metastasis of tumor cells is mediated by the reciprocal interplay between tumor cells and stromal cells and the extracellular matrix (ECM).

In this paper, we discuss the different steps of breast cancer progression and delineate the importance of the myoepithelial cell layer disruption for invasion of tumor cells. We also address the tumor promoting effect of the stromal cells in each step of the metastatic cascade of breast cancer.

## 2. Evolution of Breast Cancer

**Figure 1:**
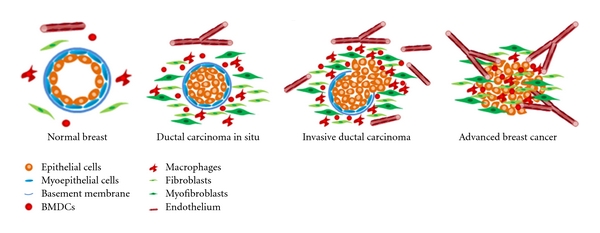
Schematic presentation of breast cancer progression accompanied with stromal cells. Normal breast duct is composed of a layer of epithelial cells and a layer of myoepithelial cells separated from the stroma by a basement membrane. Stromal cells include fibroblasts, BMDCs, endothelial cells, and other cells. Ductal carcinoma in situ (DCIS) is associated with luminal epithelial cells proliferation, and recruitment and expansion of stromal cells. In invasive ductal carcinoma, the myoepithelial cell layer is degraded with the underlying basement membrane and cancerous cells invade the surrounding microenvironment. Advanced breast cancer is associated with complete loss of myoepithelial cell layer and basement membrane, invasion of epithelial cells, proliferation of stromal cells, and angiogenesis.

The development of breast cancer involves the progression via a series of intermediate hyperplastic lesions with and without atypia (atypical ductal hyperplasia, atypical lobular hyperplasia, and usual ductal hyperplasia) followed by subsequent evolution into in situ carcinoma, for example, ductal carcinoma in situ (DCIS) and lobular carcinoma in situ (LCIS), invasive carcinomas, and metastatic cancers (Figure 1) [6–9]. In atypical hyperplasia, the breast cells are abnormal in number, size, shape, appearance, and growth pattern that may be seen as an excessive growth of cells of the ducts (atypical ductal hyperplasia) or the cells of the lobules (atypical lobular hyperplasia). In usual ductal hyperplasia, the breast tissue has an increased number of benign cells within the duct. DCIS is thought to be a precursor of invasive ductal carcinoma, in which tumor cells are confined to the lumen of the mammary duct. Lobular carcinoma in situ consists of a noninvasive increase in the cells of the milk-producing lobules of the breast. Normal breast ducts are composed of a layer of epithelial cells physically separated from the normal microenvironment by a basement membrane and myoepithelial cell layer [10, 11]. In situ carcinoma is characterized by intact myoepithelial cell layer and basement membrane, and proliferation of epithelial cells [10, 11]. When the breast tissue undergoes focal disruption of the myoepithelial cell layer and degradation of the underlying basement membrane, tumor cells invade surrounding tissues and migrate to distant organs, eventually leading to metastasis [10–12]. Despite the dramatic improvement in our ability to detect carcinomas in situ (DCIS), our understanding of the pathophysiology of this disease and factors involved in its progression to invasive carcinoma lags far behind.

## 3. Myoepithelial Cells at a Glance

The normal breast tissue is comprised of two major compartments, the epithelium and the stroma. Myoepithelial cells together with luminal cells constitute the epithelium of the ducts and of the lobule of the mammary gland. The anatomical position of myoepithelial cells between the stroma and the luminal epithelial cells from which cancer arises facilitates proper communication between both compartments. They express a number of tumor suppressor proteins (maspin), ECM structural proteins (fibronectin, collagen), proteinase inhibitors (tissue inhibitor of metallopreoteinase-1, TIMP-1), and angiogenic inhibitors (thrombospondin-1) [13, 14]. They also downregulate the expression of matrix metalloproteinases (MMPs) in fibroblasts and tumor cells [15], contribute significantly to basement membrane production, and accumulate ECM rather than degrade it [14, 16, 17]. The aforementioned functions suggest that the normal myoepithelial cell layer is a natural paracrine tumor suppressor that physically and functionally inhibits tumor growth, invasion, and angiogenesis. This tumor suppressive phenotype was identified based on the ability of myoepithelial cells to secrete paracrine factors (such as bFGF, TGF-*α*, and IL-6) that inhibit the growth and invasion of breast cancer cells in coculture assays *in vitro* [18–20]. Collective evidence suggests that myoepithelial cells also function as autocrine tumor suppressor that is supported by their resistance to transformation and their tendency to transform to tumors of low malignancy [19, 21]. Due to their tumor suppressor potential, myoepithelial cells have been referred to as the “Cinderella” of the breast [22]. Myoepithelial cells surround both normal ducts and precancerous lesions of the breast, for example, DCIS. However, DCIS myoepithelial cells differ from their normal counterparts in their ability to polarize luminal cells in three-dimensional collagen assays [16, 23], which indicates that tumor-derived myoepithelial cells are unable to transmit the necessary and correct signals to luminal cells. Moreover, myoepithelial cells isolated from normal tissue have distinct gene expression pattern as compared to DCIS myoepithelial cells. The former express high levels of laminin, tenascin, thrombospondin, cytokeratins, oxytocin receptor and tropomyosin, whereas DCIS myoepithelial cells show overexpression of proteases (cathepsins, MMP-2, and PRSS11), protease inhibitors (thrombospondin 2, SERPING1, cystatin C, and TIMP3), and collagens [24]. A common diagnostic feature of breast cancer progression from in situ to invasive tumor is the aberration of the fully differentiated myoepithelial cell layer suggesting that dissolution of the myoepithelial cell layer is an absolute prerequisite for tumor invasion. However, it is unknown what mechanisms lead to focal myoepithelial cell layer disruption and its contribution to tumor progression. Studies by Man and Sang revealed that focal myoepithelial cell layer disruptions are associated with higher leukocyte infiltration supporting the release model which is proposed by Polyak el al. to describe the role of stromal and myoepithelial cells in invasion onset [11].

## 4. Microenvironmental Influences on Breast Cancer Development

The tumor microenvironment or the stroma is composed of extracellular and cellular tissue network that surrounds and interacts with tumor cells. The cellular part includes fibroblasts, myofibroblasts, endothelial cells, adipocytes, and various immune cells [25, 26]. These cells are surrounded by an ECM that is a dynamic three-dimensional structure composed of many components including collagens, laminin, and fibronectin. The ECM is also a rich source of matrix metalloproteinases (MMPs) and soluble growth factors that affect neoplastic dissemination [27]. A specialized ECM, called basement membrane, is made of several glycoproteins and proteoglycans and separates the epithelial and endothelial cell layers from the surrounding microenvironment [28]. A well-organized basement membrane acts as a gatekeeper of invasive phenotype providing a physical support, a signaling intermediate between different compartments and a regulator of cell behavior. During tumor development, cancer cells become in direct contact with a remodeled stroma that was long considered to be a passive responder to the malignant transformation [4]. The significance of the tumor microenvironment as an active contributor in promoting and initiating breast cancer development is proposed. The difference in molecular signatures between stromal cells from tumors and normal tissues bear witness that stromal cells provide cues for tumorigenesis.

In contrast to normal fibroblasts, cancer-associated fibroblasts (CAFs) [29] enhance tumor growth and metastasis through the production of growth factors and ECM proteins and modulating immune polarization [30]. They also have different gene signatures related to paracrine signaling, transcriptional regulation, extracellular matrix, cell-cell interaction and cell adhesion/migration such as wnt1 inducible signaling pathway protein 1 (WISP1), kruppel like factor 4 (KLF4), TGF*β*2, fibulin1 (FBLN1), plasminogen activator inhibitor 2 (PAI2), and tissue plasminogen activator (PLAT) [29, 31]. Recently, Tyan et al. found that human breast cancer cells dramatically affected surrounding fibroblasts. They induced hepatocyte growth factor (HGF) production by fibroblasts to support their own growth and progression [32]. It is noteworthy to mention that a model which delineates the role of tumor microenvironment in breast cancer initiation and progression has been proposed. This model or Reverse Warburg effect suggested that tumor cells can induce an oxidative stress on neighboring fibroblasts which promotes stromal autophagy associated with Caveolin-1 loss and elevated cytokine production. This, in turn, results in production of nutrients that can nourish anabolic tumor cells [33, 34].

Tumor stroma also includes myofibroblasts, which are activated fibroblasts with *α*-smooth muscle actin (*α*-SMA) expression. In human tissue sections with invasive breast cancer, higher proportion of myofibroblasts were associated with higher-grade upregulation of Ki-67, VEGF, and bFGF, and shorter overall survival and relapse-free survival [35]. Tumor invasion and angiogenesis was shown to be promoted by *α*-SMA-positive myofibroblasts and not by *α*-SMA-negative fibroblasts [36]. Stromal myofibroblasts promote tumorigenesis of oral squamous cell carcinomas, for example, by secreting activin A [37]. In the tumor-stroma interactive microenvironment transforming growth factor-beta 1 (TGF-beta 1) promotes stromal fibroblast-to-myofibroblast transdifferentiation by modulating phenotypic and functional genes. For example, MiR-21 was recently shown to participate in TGF-b1-induced myofibroblast transdifferentiation by targeting and downregulating programmed cell death 4 (PDCD4) gene [38, 39]. Furthermore, cancer-cell-derived TGF-b release proangiogenic vascular endothelial growth factor A (VEGFA) from the myofibroblasts in esophageal squamous cell to regulate angiogenesis [40].

Implicated with angiogenesis, endothelial cells are recruited to the tumor microenvironment where they enhance neovascularization and metastasis. Adipose tissue, composed of adipocytes, has long been associated with cancer development. Coculture of adipocytes with cancer cells resulted in increased invasiveness of cancer cells and modified phenotype of the adipocytes characterized by lower lipid accumulation, decreased expression of adipocyte markers, and overexpression of proteases (MMP-11) and proinflammatory cytokines (IL-6, IL-1*β*) [41]. Il-6 depletion from adipocytes inhibited the invasion and migration of breast tumor cells [42].

The stromal compartment also contains various bone-marrow-derived cells (BMDCs) such as macrophages, mast cells, neutrophils, and lymphocytes that are recruited by the primary tumor cells to increase tumor cell migration, angiogenesis, and invasion [3]. At sites of focal disruptions of the myoepithelial cell layer in breast tissues, immune infiltrates were observed at the invasive front suggesting a potential role of these cells in malignancy and subsequent metastatic spread [10, 11].

**Figure 2:**
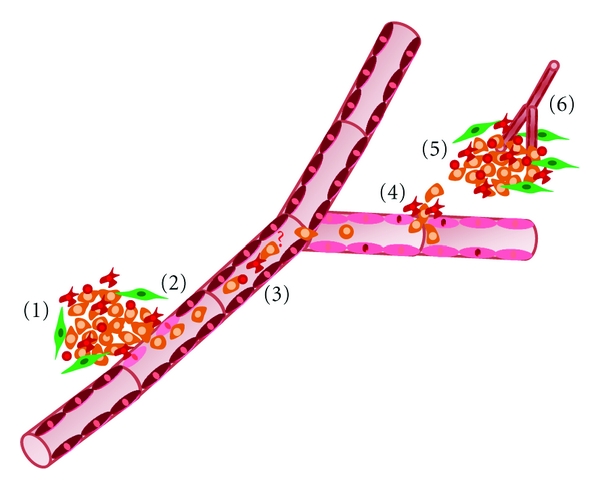
Stromal cells involved in metastatic cascade. (1) Myofibroblasts, fibroblasts, and macrophages and other BMDCs play a major role in promoting primary tumor growth. (2) Intravasation is enhanced by the paracrine interactions between tumor cells and macrophages. (3) The fusion of tumor cells and macrophages is questionable (?) and may promote the survival of the tumor cells in vasculature. (4) During extravasation, direct interaction of tumor cells and macrophages enhance tumor cell egress of the vessels. (5) BMDCs and myofibroblasts stimulate tumor cell metastatic dissemination. (6) The recruitment of endothelial cells, myofibroblasts, and BMDCs to the tumor site increases vascularization.

To metastasize, tumor cells are required to escape the primary tumor, intravasate the blood stream or lymphatic circulation, survive in the vasculature, extrude from the blood vessels or lymphatic system, arrest at distant sites, and develop into secondary mass. At each step of the metastatic cascade, stromal cells appear to be crucial players in the transition from benign to invasive and finally metastatic disease (Figure 2) [5, 43]. The metastatic cascade is a quite inefficient process meaning that failure to complete any step will quench the whole process. Until now, it is unclear which step is the key rate-limiting one that contributes to the inefficiency of the metastatic lesion. Metastatic colonization has been suggested as a major rate-limiting step because intravenous injection of cancer cells resulted in about 90% arrest and extravasation with only 0.1% of cells growing at the secondary site [44, 45]. Extravasation was also thought to be a key rate-limiting step in metastasis with highly metastatic cells extravasating faster than poorly metastatic ones. However, a study by Koop et al. showed that extravasation is independent of the metastatic ability and highly metastatic ras-transformed cells extravasate at the same rate of control fibroblasts [46]. Similar to extravasation, intravasation was proposed to be a major rate-limiting step. *In vivo* data showed that intravasation can be a major rate-limiting step in the metastatic cascade. Wyckoff et al. showed that metastatic cells exhibited a faster entry into the vasculature than poorly metastatic cells. Moreover, Zijlstra et al. quantitatively evaluated the rate limiting steps in HEp-3 and HT-1080 human tumorigenic cells. The authors found that HEp-3 cells had higher metastatic rate than HT-1080 cells due to the lower efficiency of the latter cells in intravasation and metastatic colonization [47]. Collectively, intravasation and growth at secondary sites represent major rate-limiting steps in the metastatic cascade. However, the rate-limiting step may vary depending on the tumor type [45].

### 4.1. Primary Tumor Growth

Under normal physiological conditions, the surrounding microenvironment imposes proper tissue architecture maintained by basement membrane alignment and intercellular communication [3]. This interplay between epithelial cells and surrounding stroma maintains organ homeostasis that serves as a protective constraint against malignant transformation. During tumor development, cancerous cells circumvent the normal controls regulating the activity of ECM proteases. In response to these proteolytic enzymes, the basement membrane undergoes gradual degradation and structural changes causing violation of normal tissue boundaries and conduits for malignant cell egress. To invade, tumor cells should lose cell-cell and cell-ECM interactions mediated by integrins and cadherins. Invasion is also accompanied by proteolytic degradation of surrounding tissue mediated by proteases, motility of tumor cells mediated by chemokines and growth factors, and recruitment of stromal cells.

 Fibroblasts play an important role in cancer progression. They are primarily responsible for the synthesis, deposition, and remodeling of the basement membrane and ECM through the production of collagen (I, III, IV, and V), fibronectin, and laminin. They are also an important source of paracrine growth factors (HGF, EGF, FGF2, and TGF*β*), proteolytic enzymes (MMP-1, MMP-7), and cytokines (IL6, CXCL12) that can affect cell proliferation, survival, morphology, and death [48, 49]. During tumorigenesis, fibroblasts maintain an activated phenotype characterized by the expression of *α*-smooth muscle actin (*α*-SMA) and are referred to as carcinoma-associated fibroblasts (CAFs) or myofibroblasts [49]. In breast carcinomas, about 80% of stromal fibroblasts acquired the CAF-activated phenotype [48]. During tumor development, CAFs exhibit a higher proliferation index and become the predominant cell population in the stroma. Once the basement membrane is degraded, CAFs are accumulated causing expansion of tumor stroma and increased deposition of ECM through expression of stress fibers and *α*-SMA. This phenotype is termed desmoplasia and is associated with recruitment of inflammatory cells and activation of angiogenesis [50]. Several studies demonstrated a direct involvement of fibroblasts in initiation of cancer. In a xenograft mouse model, Kuperwasser et al. showed that the upregulation of transforming growth factor-*β* (TGF*β*) or hepatocyte growth factor (HGF) in mouse fibroblasts stimulated the initiation of benign and malignant lesions in the breast epithelium [51]. In addition, a gene expression profiling study of all cell types in normal and neoplastic breast demonstrated that overexpression of CXCL12 in myofibroblasts was correlated with epithelial cell proliferation and invasion [23]. Another study of xenograft mouse model coinjected with MCF-7 breast cancer cells and CAFs or normal fibroblasts showed that xenografts infused with CAFs had enhanced growth than xenografts injected with normal fibroblasts [52]. To reconcile, these data reveal the major role of CAFs in tumor initiation and progression.

An initial reaction of the host to the tumor development is the recruitment of leukocytes and subsequent local inflammation [53]. As a physiological response to tissue injury, inflammatory cells are recruited to the injured site to support tissue repair and remodeling through the production of growth factors and cytokines such as TNF-*α*, CCL2, CXCL8, CCL5, TGF-*β* and so forth. In normal conditions, the inflammatory response is resolved once the tissue is repaired [54, 55]. However, the tight regulation of inflammation is overridden during malignant transformation recalling the historic view of tumors as “wounds that never heal” [56]. Persistent inflammatory response characterized by activated leukocytes and secretion of several cytokines and chemokines (including tumor necrosis factor (TNF), interleukins, and interferons) elaborates the formation of tumor-promoting microenvironment. Such protumor stroma is incentive not only for primary tumor development but also for metastatic dissemination into systemic circulation.

Neoplastic transformation is regulated by dynamic reciprocity between epithelial cells, activated stromal cells and ECM components. If the changes in the microenvironment occur prior or concomitant with epithelial cell changes is still debatable. Recently, a gene expression profiling study on laser captured breast stroma and epithelia showed 90% change in the stromal gene expression at the transition from normal to DCIS, and only 10% stromal alterations in DCIS compared to invasive disease [57, 58]. Another study used mRNA in situ hybridization of breast tissue with different stages of cancer to examine the expression of angiogenic factors and stromal components. A similar expression profile was observed in carcinoma in situ, invasive cancer, and metastatic disease [59]. These studies suggest that stromal changes induced by the emerging epithelial lesions precede invasion and that cancer cells invade into an abnormal breast microenvironment with growth-promoting effects.

### 4.2. Intravasation

Intravasation or penetration of tumor cells into the vasculature involves the movement of cancer cells through the ECM, the basement membrane, and finally through the endothelium of the blood vessel or lymphatic duct. The underlying molecular mechanisms governing intravasation are not clear as all studies have focused on later steps in the metastatic cascade. Detailed evaluation of all steps in tumor metastasis is crucial to understand the cellular mechanisms controlling neoplastic dissemination. In a murine breast cancer model, Yang et al. found the transcription factor, Twist, as a key regulator of metastasis [60]. It augments epithelial-to-mesenchymal transitions and promotes the rate of hematogenous intravasation. Another possible mechanism that executes the migration of tumor cells across the vessel wall was shown by intravital imaging studies of experimental mammary carcinomas [61, 62]. These studies showed that carcinoma cells intravasate through the blood vessels due to chemoattractive gradients generated by perivascular macrophages that are recruited by the tumor cells to the injured site [61]. In breast carcinomas, macrophages and cancerous cells form a paracrine loop involving epidermal growth factor (EGF) and colony stimulating factor-1 (CSF-1) to augment chemotaxis and intravasation [63]. EGF produced by macrophages promotes migration of neoplastic cells into hematogenous vasculature through its interaction with EGF receptor expressed on breast cancer cells. Tumor cells, in turn, express CSF-1 which acts as a potent chemoattractant for CSF-1 receptor positive macrophages [64]. This crosstalk lends credence to the collaborative work between tumor microenvironment and neoplastic cells at the site of intravasation.

### 4.3. Survival in Vasculature

Once malignant cells have invaded the angiogenic vasculature, they are subject to harsh microenvironment characterized by hemodynamic shear forces, surveillance of immune cells, and lack of substratum [43]. To bypass these perils, tumor cells use platelets as a shield. Through their tissue factor, tumor cells bind coagulation factors (VIIA and X) on the platelets creating an embolus that arrests in the capillaries [65, 66]. These aggregates protect the cancerous cells from immune-cell-mediated lysis and decrease the shear forces of the blood circulation, thus increasing their survival, arrest, and extravasation [67]. Whether macrophages can infer protective effects on neoplastic cells in the bloodstream as they do during invasion and intravasation is not yet known. To explain the metastatic phenotype, Pawelek and Chakraborty proposed the fusion of macrophages or other BMDCs with cancer cells forming a hybrid capable of surviving in the circulation and homing to secondary sites [68]. Should the fusion theory be accepted in human cancers, BMDCs would have a beneficial role in the survival of tumor cells in the vasculature.

### 4.4. Extravasation

After survival and arrest in the circulation, tumor cells must escape out of the blood and lymphatic vessels in a process known as extravasation. To do so, tumor cells induce disruptions in the endothelial junctions that allow tumor cells to bind to the subendothelial ECM and extrude into target organs. Vascular permeability is mediated by activated Src kinases in endothelial cells, which once exposed to vascular endothelial growth factor (VEGF) from tumor cells promotes endothelial retraction, resulting in movement of cancer cells towards surrounding tissues [69]. To identify the role of macrophages in metastasis, Qian et al. used an animal model of breast cancer metastasis and an intact *ex vivo* lung imaging system to show that modified host macrophages are required for proper metastatic seeding and growth [70]. The authors found that macrophage ablation dramatically decreased the number of tumor cells observable in the lungs [70]. Moreover, lung-resident macrophages were visualized to physically interact with tumor cells as soon as they extravasate through the vessel walls, thus promoting the rate of extravasation and metastatic dissemination [70]. Immunophenotyping of metastasis-associated macrophages showed distinct profile from lung resident macrophages. The prometastatic macrophages are characterized by cell surface expression of CSF1R, CD11b, F4/80, with high levels of CCR2, CX3CR1, and VEGFR, absence of Gr1 and low CD11c [70, 71].

### 4.5. Metastatic Site

Metastasis is regulated not only by changes in tumor cells but also by reciprocal interactions with the surrounding microenvironment. In his “seed and soil” hypothesis, Paget proposed that tumor cells or “seeds” can only colonize microenvironments or “soils” that are compatible with their growths [72]. For example, breast cancers metastasize to lungs, bone, liver, and brain, whereas advanced prostate cancers colonize the bone as the predominant site [45]. Since the circulatory patterns provide only partial explanation for the tissue tropism aspect of metastasis, several molecular and cellular mechanisms have been proposed. An emerging paradigm suggests that primary tumor cells may secrete factors capable of inducing a fertile microenvironment, termed premetastatic niches, that favors the seeding and proliferation of metastatic cells at unique sites [73]. For example, (ADAMTS1) and matrix metalloproteinase-1 (MMP1) participate in a paracrine signaling cascade that includes the release of metastasis membrane-bound epidermal-growth-factor (EGF-) like growth factors, amphiregulin (AREG), heparin-binding EGF (HB-EGF), and transforming growth factor alpha (TGF alpha) from tumor cells resulting in a downregulation of osteoprotegerin expression in osteoblasts and therefore modulating brain microenvironment in favor of osteoclastogenesis and bone metastasis [74]. Recent work have identified the following mediators of extravasation and brain colonization: the cyclooxygenase COX2, the epidermal growth factor receptor (EGFR) ligand, HBEGF, and the alpha 2,6-sialyltransferase, ST6GALNAC5, which were found to enhance breast cancer passage through the blood brain barrier and to facilitate their adhesion to brain endothelial cells [75]. EGFR ligands and COX2 are also linked to breast cancer infiltration of the lungs [76, 77]. Recently, breast cancer cells infiltrating the lungs were shown to support their own metastasis-initiating ability by expressing tenascin C (TNC). TNC is an extracellular matrix protein of stem cell niches that promotes the survival and outgrowth of pulmonary micrometastases via upregulation of stem cell signaling components, musashi homolog 1 (MSI1), and leucine-rich repeat-containing G protein-coupled receptor 5 (LGR5) until the tumor stroma takes over as a source of TNC [78]. Through the production of growth factors (VEGFA) and chemokines (S100A8, S100A9), tumor cells induce the recruitment of BMDCs, and endothelial progenitor cells to the premetastatic niche [79]. The bone marrow progenitors are VEGF receptor-1 (VEGFR1) positive and can migrate and proliferate in response to tumor-derived VEGF [79]. These VEGFR1^+^ cells also express integrin VLA-4 and tend to form clusters induced by integrin-fibronectin interactions, the latter of which is synthesized by resident fibroblasts [73]. Several other molecules have been implicated in preparing the premetastatic niche and increasing metastasis. Matrix metalloproteinase-9 (MMP-9) secreted by BMDCs degrades the basement membrane and liberates the matrix-sequestered VEGFR1 ligand, the VEGFA, promoting the homing of more VEGFR1^+^ cells into the niche [80]. Through the production of VEGFA, TGF*β*, and tumor necrosis factor-*α* (TNF*α*), tumor cells enhance the expression of the chemoattractants S100A8 and S100A9 in lung endothelium and myeloid cells, which in turn promote tumor cell homing and adhesion to the metastatic site [81, 82]. The role of activated fibroblasts in metastasis has been revealed in some studies. Fibroblast-specific protein-1 (FSP-1/S100A4), a fibroblast-specific marker, is highly expressed on tumor-associated fibroblasts and is released upon stimulation of fibroblasts by tumor cells. Mice deficient in FSP-1 exhibited a significant reduction in tumor growth and metastasis [83]. Injection of FSP-1-positive fibroblasts into these mice restored the ability of mammary adenocarcinoma cells to develop tumors and generate metastasis suggesting a potential role of tumor-associated fibroblasts in the metastatic dissemination. Another study found that only metastasized melanoma cells were affected by fibroblasts suggesting that fibroblasts might be important in creating the permissive soil that supports tumor cell growth at distant sites [84]. Recently, O'Connell and colleagues found that S100A4^+^ fibroblasts provide the proper metastatic niche to support metastatic colonization. Through production of VEGFA and tenascin-C, fibroblasts can promote angiogenesis as well as provide protection against apoptosis, respectively [85].

Following the implantation of tumor cells, persistent growth of metastasis is maintained by the establishment of sufficient blood supply capable of providing the necessary oxygen, growth factors, nutrients, and metabolites. Blood vessels are composed of vascular basement membrane, endothelial cells and specialized smooth muscle cells, the pericytes [28]. The induction of a tumor vasculature termed the angiogenic switch requires basement membrane assembly, recruitment and proliferation of endothelial precursors, and pericytes attachment [86]. Initially, the vascular basement membrane is degraded by several MMPs produced by stromal cells, endothelial cells, or tumor cells [87]. This basement membrane degradation causes the release of endothelial cells to migrate and proliferate, the liberation of matrix-sequestered growth factors such as VEGF, basic fibroblast growth factor (bFGF), and platelet-derived growth factor (PDGF) and the disassembly of the pericytes that line the blood vessels [87]. In response to VEGF, VEGFR2^+^ endothelial progenitor cells are recruited to the metastatic site through VEGFA signaling to contribute to vessel formation [88]. Analysis of these progenitor cells shows upregulation of several angiogenic molecules (VEGF, FGF, PDGF, CXCL1, etc.) that further bolster local angiogenesis and subsequent metastatic colonization [86]. Moreover, VEGFR1^+^ BMDCs has been shown to produce several angiogenic factors and are required to provide stability to the neovessels [89]. Inhibition of VEGFR1^+^ BMDCs either during primary tumor or after the formation of premetastatic niche caused the prevention of endothelial cell migration and metastasis [79]. Thus, the recruitment of VEGFR2^+^ endothelial progenitor cells into vessels requires the incorporation of VEGFR1^+^ BMDCs to support neovascularization [60, 73]. Tumor angiogenesis is also regulated by several immune cells [90]. Macrophages, for example, are a good source of angiogenic factors such as VEGF and MMP-9 [91]. In a mouse model of highly aggressive metastatic mammary carcinoma, Lin and Pollard found that tumor-associated macrophages may provide essential cues to press the angiogenic switch [91]. Colony stimulating factor-1 (CSF-1) deletion caused failure in macrophage homing to the malignant stroma that was associated with attenuated angiogenic responses, decreased neoplastic progression and inhibition of pulmonary metastasis. Moreover, CAFs have been shown to be actively involved in boosting tumor angiogenesis. The coinjection of CAFs and MCF-7 breast cancer cells into nude mice resulted in the recruitment of bone-marrow-derived endothelial progenitors in response to CAF-derived stromal cell derived factor (SDF1/CXCL12) stimulating angiogenesis and tumor formation [52]. Another proangiogenic mechanism of CAFs involves the release of several factors such as VEGF and FGF which can positively contribute to vascularization [48].

## 5. Perspectives

It is evident that metastasis is a multistep process where each stage requires an intricate interplay between cancerous cells and cells of the microenvironment. This tumor-host crosstalk supports the notion that cotargeting cancer cells and tumor stromal cells will be a viable approach for mammary cancer prevention and treatment. Researchers are dedicated to explore the stromal cells as an effective target for anticancer therapeutics. Such host-targeted therapies should be directed towards BMDCs, fibroblasts, and endothelial cells that home to the metastatic site to support tumor dissemination and outgrowth. It is important to inhibit the mobilization and proliferation of the stromal cells and to disrupt tumor-stroma interactions mediated by paracrine factors. To achieve these goals, several agents have been investigated and they fall into several categories including protease inhibitors (e.g., MMP inhibitors), antiadhesive molecules (e.g., anti-integrin peptides or antibodies), signal pathway modulators (e.g., tyrosine kinase pathway inhibitors), antifibrotic drugs (e.g., pirfenidone), and antiangiogenic molecules (e.g., VEGF and bFGF antagonists) [92, 93]. Clinical trials using MMP inhibitors (MMPIs) were disappointing for several reasons. The trials were conducted only on patients with advanced disease, and MMPIs used were broad spectrum MMPIs which can block bad MMPs as well as good ones. Avastin/bevacizumab, a monoclonal antibody targeted against all isoforms of VEGF-A, has recently been withdrawn from the FDA list for the treatment of breast cancer [94]. Unlike trastuzumab which is a HER-2-targeted antibody, avastin delayed tumor progression with no improvement in overall survival. This was accompanied by adverse side effects including hypertension, neuropathy, and infection [95]. Recently, a variety of studies have been conducted to target the tumor microenvironment. Wu et al. have shown that targeting Galectin-1 to significantly inhibit CAF-conditioned medium-induced tumor cell migration and invasion in oral squamous cancer cells (OSCCs) resulting in a reduced metastasis *in vivo* [96]. It is followed that Galectin-1 downregulation reduces the production of monocyte chemotactic protein-1 (MCP-1/CCL2) which promotes the migration of OSCCs by binding to CCR2 receptor. Blocking the interaction between MCP-1 and CCR2 abolishes migration. Moreover, Kim et al. have proposed to target myofibroblasts overexpressing laminin-332 which caused the formation of the dense fibrosis via desmoplastic reaction during epithelial to mesenchymal transition (EMT) [97]. This alteration of the tumor microenvironment preceded tumor invasion and was found in invasive ductal carcinoma. Targeting of laminin-332 overexpressing myofibroblasts was supposed to prevent the formation of the dense fibrosis, thus inhibiting the invasion-friendly stromal alteration. It is important to note that Angiotensin-(1–7), an endogenous 7-amino acid peptide hormone of the renin-angiotensin system, has been shown to target the tumor microenvironment to inhibit CAF growth and tumor fibrosis [98]. Additionally, Liu et al. proposed the targeting of the coagulation cascade that is activated in the tumor microenvironment and presented preclinical data targeting tissue factor (TF), an enzyme cofactor in activating coagulation that plays a critical role in tumor growth [99]. TF inhibition by TF:FVIIa inhibitor led to growth retardation in tumor models. Coenegrachts et al. demonstrated that the selective neutralization of host-derived bone-derived placental growth factor (PlGF) by anti-mouse alphaPlGF reduced the engraftment of tumor cells in the bone, inhibited their interaction with matrix components, reduced the incidence, number, and size of bone metastases, and preserved bone therefore inhibiting both the progression of metastasis and the settlement of tumor in the bone [100]. Truitt et al. have shown the role that Eph receptor tyrosine kinase EphB6 plays in suppressing cancer invasiveness through c-Cbl-dependent signaling, morphologic changes, and cell attachment and that its targeting might enable the regulation of both cell attachment and migration [101]. These stroma-targeted therapies combined with antitumor approaches will be translated into a double-edged sword that cancerous cells will not easily survive. These therapeutic approaches require a full understanding of the cellular and molecular mechanisms governing the tumor-host interactions, accompanied with the development of new mouse models and intravital imaging techniques. Once accomplished, cancer patients will experience better survival rates and quality of life.
